# On Power-Off Temperature Attacks Potential Against Security Sensors

**DOI:** 10.3390/s25061912

**Published:** 2025-03-19

**Authors:** Maryam Esmaeilian, Vincent Beroulle, David Hély

**Affiliations:** LCIS, Grenoble INP, University Grenoble Alpes, 26000 Valence, France; maryam.esmaeilian@lcis.grenoble-inp.fr (M.E.); vincent.beroulle@lcis.grenoble-inp.fr (V.B.)

**Keywords:** hardware security, fault injection attack, power-off attack, temperature attack, secure circuit, delay-based detectors

## Abstract

Embedded systems can be targeted by fault injection attacks (FIAs), which enable attackers to alter the system specified behavior, potentially gaining access to confidential information or causing unintended outcomes, among other effects. Although numerous security sensors and attack detectors have been proposed in the literature to detect different sources of FIAs, it is crucial to ensure that these mechanisms themselves have not been tampered. Hence, the integrity of these detectors is critical in maintaining the security of embedded systems. This study focuses on evaluating the robustness of delay-based digital detectors against a new type of FIA called power-off temperature attack (POTA). POTA occurs when the chip power is turned off, rendering the detectors inactive and allowing the attackers to bypass them. After a POTA, the circuit or its detectors may not function properly when the power is restored, potentially allowing other attacks to go undetected if the detectors are less sensitive. This study implements two attack detectors on Xilinx Artix-7 FPGAs and investigates the impact of heating cycles on theses detectors’ characteristics when the FPGA is in different states, including power-off, power-on, and inactive modes (such as clock-freezing mode). Our experiments reveal that heating cycles in power-off or inactive modes can alter the FPGA component delays and reduce the accuracy of its detectors, which highlights the vulnerability of these systems to POTA and potential risks to embedded system security.

## 1. Introduction

Electronic devices are increasingly employed in security applications, such as authentication applications. As a consequence, these devices are the target of many different attacks aiming at either modifying their normal behavior or revealing secret data, such as cryptographic keys. Due to the nature of their applications, such devices are particularly vulnerable to physical attacks, where the attacker can leverage physical access to a device and further perform a so-called hardware attack, such as a side channel attacks (SCA) [1] and fault injection attack (FIA) [2].

SCAs represent a class of security vulnerabilities that exploit unintended information leakage from a device during its operation. SCAs focus on the physical characteristics of a device, such as its power consumption, execution time, electromagnetic emissions, or even sound emissions. By analyzing these side-channel signals, attackers can glean valuable information about the device’s internal processes, leading to the extraction of sensitive data or cryptographic keys. The fundamental premise behind SCA is that the behavior of a device, even one designed securely, can inadvertently reveal subtle clues about the computations it performs. For example, when a device performs cryptographic operations, the electrical power it consumes might vary depending on the specific operations being executed. Similarly, the time it takes to complete certain tasks can also provide hints about the data being processed.

Unlike SCAs, where the attacker does not affect the normal operation of the system, FIAs involve intentionally disrupting the normal operation of the system by injecting faults. These faults can stem from various sources, such as altering the system’s component electrical parameters. The result is a deviation from expected behavior, potentially granting attackers unauthorized access or exposing vulnerabilities. In this study, our main focus is on these FIAs.

The techniques for FIAs are becoming increasingly advanced and powerful. This development causes an important concern and a threat to systems where security plays a fundamental role. So, numerous security experts and designers are actively developing protection and detection mechanisms to mitigate the risks associated with such attacks. One of these mechanisms is the use of detectors (or sensors) to detect FIAs. These detectors, which can be digital or analog, can detect FIAs [3,4]. Recent works have mostly focused on digital detectors because they can be easily calibrated and placed close to security primitives, such as PUFs and encryption cores. For example, in [5] a digital detector is presented to detect an FIA based on electromagnetic radiations. Furthermore, digital detectors introduced in [6,7] can detect clock glitching and voltage glitching attacks, respectively.

Detectors are used to protect the system from FIA. However, it is crucial to ensure that these detectors themselves are protected against potential FIAs. There is a bunch of research work on the vulnerability of detectors [8] to various types of FIAs as well as methods for protecting them. However, all previous studies evaluating detectors against FIAs have assumed that the detectors are connected to the power supply. However, none have assessed their effectiveness against FIAs when the power is off.

Recent research has demonstrated that laser fault injection (LFI) can be performed even when a device is not powered [9]. This study targets the Flash memory of 32-bit microcontrollers, showing that laser exposure can introduce persistent faults without requiring real-time synchronization between the attacker and the system. These findings highlight the increasing sophistication of FIA techniques and their potential impact on security. However, while prior works have investigated attacks on unpowered devices, they have not specifically examined their effects on FIA detectors. Our study addresses this gap by evaluating how power-off temperature attacks (POTAs) impact digital FIA detectors. This new type of FIA, which we call a POTA, is performed when the target device is not connected to any power supply. The characteristics of the detectors can be changed by an attacker without being detected, as the detectors are off. Such changes can adversely affect the key detector features, such as the detection thresholds, leading to a modification in the false-positive and false-negative detection rates. Alterations can affect detector accuracy, while an increase in the false-negative rate may lead to security risks and grant unauthorized access to the system to attackers. Unlike [9], which focuses on modifying stored memory contents, we analyze how temperature-induced variations can alter detection thresholds and compromise the reliability of security mechanisms.

The objective of this work is to evaluate potential attacks on FIA detectors while the system is powered off. Our main contributions are as follows: First, we introduce the concept of a POTA and demonstrate its impact on digital FIA detectors. Second, we investigate how high temperatures induced by external heating can alter detector characteristics and compromise detection reliability. Finally, we conduct experimental validation using a Ring Oscillator(RO) and a digital detector to analyze the effects of LFI and aging in power-off attacks. Our study focuses on the detector proposed in [10] and a RO to assess the impact of LFI and aging attacks when the target device is turned off. To simulate these attacks, we use overheating to emulate the effects of LFI and aging, as overheating cycles can induce permanent variations in the detector’s characteristics. These persistent changes can be critical, particularly in cases where false negatives occur in FIA detection.

The general aim of this study is how heating (when the power is off) impacts the properties of simple digital detectors. To conduct this experiment, we subjected the chip to various periods of heating, as certain FIAs like LFI can lead to temperature elevations. By heating the circuits under study, we were able to evaluate the impact of the FIA on the chip’s properties and determine its effects on the detection threshold.

The rest of this paper is organized as follows: In Section 2, we review the state-of-the-art methods related to our work. In Section 3, the structure and methodology of this study will be explained. Experimental results are presented in Section 4. In Section 5, we discuss the results. At the end of this paper, in Section 6, we conclude and provide perspectives for future works.

## 2. Related Work

### 2.1. Digital and Analog FIAs Detectors

Several methods have been introduced in the literature to protect devices against FIAs. These techniques can be based on redundancy at different levels or on techniques based on sensors’ use. The advantage of redundancy is that it allows for faults to be detected independently of the FI technique. However, the main disadvantage of this method is that this method cannot capture all possible faults [11]. The second technique is to use fault detection sensors, also known as detectors. Detectors can be divided into two categories, digital detectors and analog detectors; the analog type, as its name suggests, uses analog sources to detect FIAs. In [12], a type of analog detector is proposed that uses a time-to-digital converter to detect FIAs. These types of detectors are much more difficult to calibrate than digital ones because they use analog sources; on the other hand, they require more power consumption. Therefore, digital detectors are widely used today. Different digital FIA detectors are proposed in the literature [13,14]. One of the most popular designs, named delayed-based detector, has been suggested in [15]. This detector is based on the timing constraints of the synchronous circuits, as shown in Equation (1). To guarantee that synchronous circuits operate correctly, the clock period ($T_{Clock}$) must be greater than the sum of the propagation delay ($T_{PropagationDelay}$) and the setup time ($T_{Setup}$); otherwise, the circuits do not have enough time to perform its operations.

(1) $$T_{\mathrm{Clock}}\geq T_{\mathrm{PropagationDelay}}+T_{\mathrm{Setup}}$$

Delayed-based detectors can detect various FIAs, such as clock glitching, under-powering or overheating [16,17,18].

**Figure 1:**
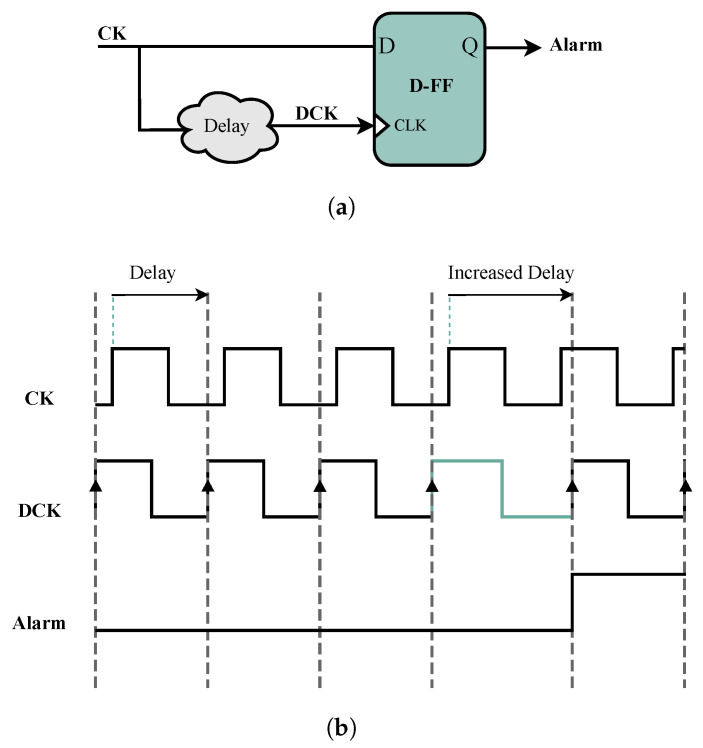
(**a**) Schematic diagram and (**b**) waveforms of the delay-based detector proposed in [15].

Figure 1 illustrates how this detector compares the delayed clock signal (denoted DCK) with the primary clock signal using a D Flip Flop (D-FF). If there is a malfunction (e.g., delay variations or clock period increases), the alarm is activated. While this detector is simple and efficient against certain FIAs, it is less effective for attacks with localized effects, such as laser or electromagnetic FIAs. This limitation exists because detectors can detect violations from global sources. However, using a network of these detectors can improve the detection of localized attacks [19]. Additionally, this type of detector can detect FIAs that increase the clock period or propagation delay but is unable to detect attacks that decrease the clock period [19]. Accordingly, other designs have been introduced to improve the detection rates against FIAs. The next category of proposed detectors is based on RO. ROs can be implemented using a closed chain of odd-numbered inverters [19]. In this structure, an RO alternates between zero and one. Thus, it can be a frequency generator whose output frequency depends on the number of inverters and propagation delays.

The implemented ROs can be used in a detector’s design. For instance, as shown in Figure 2 from [20], this detector consists of two high- and low-phase circuits, which are used for the one- and zero levels of the clock signal, respectively.

Each of these circuits counts the number of RO oscillations at each clock level and then compares them with a constant value. In normal mode, the number of RO oscillations is always the same, but when under attack, the number of RO oscillations changes and is not equal to the constant value, which triggers the alarm. Since this detector uses two separate circuits for the zero/one levels of the clock, it can detect attack attempts at each level of the clock and thus can speed up the fault detection. Furthermore, in order to detect an attack, it completes a comparison with a constant value. Therefore, it is capable of detecting attacks that can decrease and increase the clock period, exposing a higher accuracy than the delay-based detector that is proposed in [15]. In [14], the authors evaluated glitch detection circuits against FIAs. Their experiments revealed significant weaknesses, as they successfully bypassed the detectors using four distinct glitching attacks. The effectiveness of these detectors was shown to depend heavily on internal parameters and the techniques used to attack the circuit. This suggests that FIA detectors may also be vulnerable to other types of attacks. In our work, we specifically evaluate the robustness of these detectors against power-off temperature attacks, aiming to further investigate their potential weaknesses under varying environmental conditions. In this study, we aim to evaluate the effects of POTAs on delay-based detectors. These detection circuits have been extensively studied in the literature and are widely deployed to counter various FIA methods. Indeed, several FIA techniques induce timing violations. Notably, timing violation detection circuits have been proposed as effective countermeasures against attacks that exploit timing anomalies, including under-powered voltage glitching [21] and electromagnetic FIAs [15]. Consequently, any attack detector designed to identify other threats could also be vulnerable to POTAs if it relies on delay-based principles. These detectors are widely adopted due to their capability to detect a broad range of FIA techniques. More recently, Intel announced the integration of such detectors into Intel Core processors [22]. Given the growing reliance on these mechanisms, understanding their potential vulnerabilities to POTAs is crucial for enhancing security.

### 2.2. Aging Effects

Aging is a critical factor to consider in our work, as it directly impacts the long-term performance and reliability of FIA detectors. Over time, aging mechanisms can degrade transistors [23,24,25], potentially increasing the vulnerability of these detectors. The following four primary mechanisms of aging are commonly referenced in the literature [26].

#### 2.2.1. Bias Temperature Instability (BTI)

**Figure 3:**
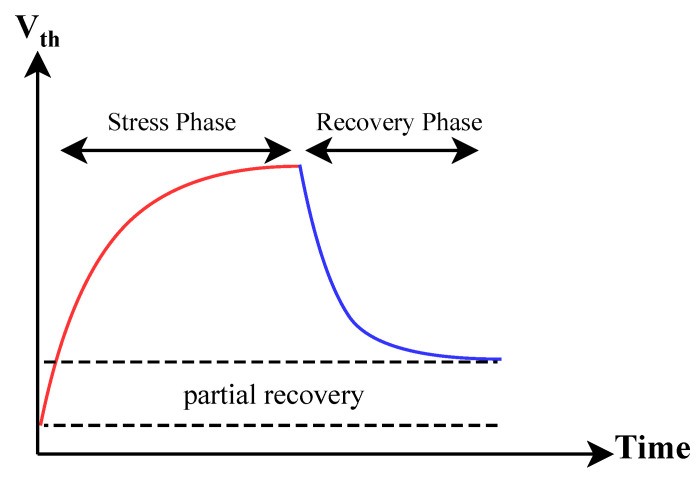
BTI has two phases: the stress phase and the recovery phase.

Bias temperature instability (BTI) is an aging mechanism that affects the reliability of transistors in integrated circuits. It occurs because of prolonged exposure to electrical stress and elevated temperatures during device operation. BTI leads to a gradual increase in the transistor’s threshold voltage and a decrease in its performance. Over time, this degradation can result in reduced speed and power efficiency in electronic devices, affecting their overall lifespan and reliability. BTI consists of two main phases, as shown in Figure 3:

- **Stress Phase:** BTI begins when a transistor is under constant electrical stress during operation. This stress gradually affects the transistor’s performance, mainly by increasing its threshold voltage.
- **Recovery Phase:** When stress is removed, the transistor might partially recover its original performance, but it may not fully return to its initial state, as shown in Figure 3. The recovery phase follows the stress phase in BTI.

#### 2.2.2. Hot Carrier Injection (HCI)

HCI is another aging effect that affects the reliability of transistors. It occurs when high-energy electrons or holes are injected into the gate oxide of the transistor under high-voltage conditions. This injection can create traps or defects in the oxide layer, which degrades the transistor’s performance and can lead to threshold voltage shifts. HCI is more pronounced in transistors with smaller feature sizes, as they are more susceptible to higher electric fields.

#### 2.2.3. Time-Dependent Dielectric Breakdown (TDDB)

Time-dependent dielectric breakdown (TDDB) refers to the gradual weakening of the insulating material in electronic devices over time due to sustained electrical stress and voltage. This degradation can lead to the formation of microscopic defects, which may result in electrical leakage or short circuits, ultimately reducing the reliability and operational lifespan of the device.

#### 2.2.4. Electromigration

Electromigration occurs when metal atoms in electronic components gradually move due to high current and heat. Over time, this can weaken connections and lead to electrical failures, affecting the reliability of the device.

The aging effects on transistors can result in a variety of issues, such as increased power consumption, reduced switching speed, and diminished overall circuit reliability. Between all aging mechanisms, BTI and HCI have a greater effect on the performance of the transistor [27].

In this section, we discussed previous works related to countermeasures against FIAs, particularly focusing on digital detectors. Additionally, we discussed aging and its effects. The two main factors contributing to aging in transistors are temperature and electrical stress. As explained earlier, our project aims to assess the impact of LFI and aging attacks on FIA detectors and a basic RO. One of the effects of LFI is an increase in temperature at the targeted location of the chips. High temperature is also a method to accelerate aging. Therefore, in our evaluation, we use high temperature to simulate the effects of LFI and aging attacks.

## 3. Methodology

In this section, our goal is to explain the detection principles of two device under tests (DUTs) and the method of our experiment. As explained in the previous section, our goal in this study is to evaluate the effect of LFI on the sensitivity of the detector when the power of the device is turned off. As recalled earlier, heating (due to several possible sources) is a major source of aging for integrated circuits; in the rest of this study, we consider heating (without considering the source, which can be a laser or simply an oven) as a characterization means to analyze the effect of power-off attacks.

### 3.1. Device Under Test (DUT)

In this case study, we chose two DUTs for our experiment: a delay-based detector and a RO-based detector.

#### 3.1.1. Delay-Based Detector

**Figure 4:**
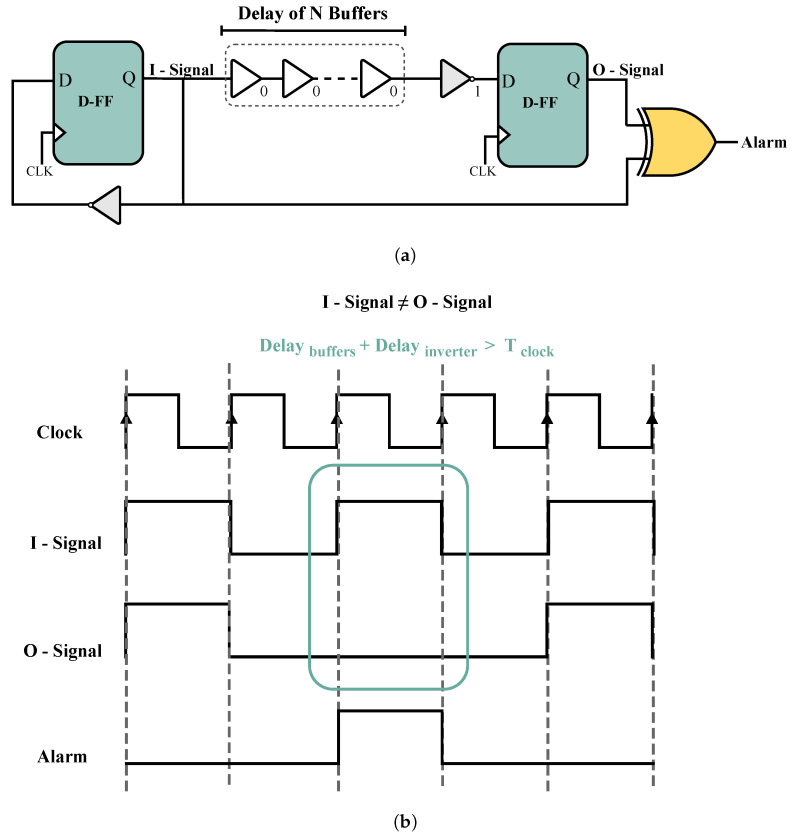
(**a**) Schematic and (**b**) waveforms of the delay-based detector proposed in [10].

For the first DUT, we selected the delay-based detector that was proposed in [15] because this type of detector is less complex to implement than other types. Figure 4a illustrates the delay-based detector, which operates by keeping the alarm inactive while the output of the two flip-flops (represented as the I-Signal and O-Signal in Figure 4a are equal. However, when these two signals are complementary, the alarm will be activated. In fact, if the delay of the buffer chain exceeds the clock period, the I-Signal may not arrive at the second flip-flop input in time, causing the outputs of the two flip-flops to differ and trigger the alarm. The number of buffers should be selected carefully to ensure that their delay is sufficiently close to the clock period. This will ensure that the alarm remains inactive in normal operation but becomes active in the event of an attack on the circuit. As shown in Equation (2), as long as this equation is true, the alarm is not activated. FIAs, such as heating and laser, can increase the delay of logic gates, thereby modifying Equation (2) and triggering this detector.

(2) $$T_{clock}\geq {Delay}_{\left(buffers+inverter\right)}+T_{setup}$$

The sensitivity of the detector increases as the delay caused by the buffers approaches the clock period. However, this also increases the likelihood of false positives. Therefore, there is a trade-off between improving the accuracy of the detector and increasing the number of false positives.

As mentioned earlier, selecting the optimal number of buffers is critical for implementing this detector, since we need to choose the number of buffers whose delay is close to the clock period.

#### 3.1.2. Ring Oscillator (RO)

**Figure 5:**
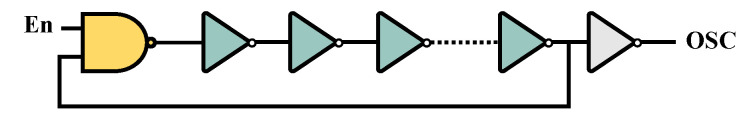
Ring oscillator (RO).

As many detectors rely on RO, the second device under test (DUT) is a basic RO design. Furthermore, compared to delay-based detectors, RO-based detectors offer a better understanding of how power-off attacks function (Figure 5).

### 3.2. Our Test Scenarios

To improve the precision of assessing the impact of POTAs on our DUTs, it is necessary to define several test scenarios. Hence, we incorporate a condition on power and an additional condition on temperature. In the subsequent section, we will examine the aforementioned conditions and their potential contribution to our evaluation process.

#### 3.2.1. Temperature Condition

To have a comprehensive comparison to evaluate the effect of heating, we have three temperature conditions.

1. **First Condition:** Constant Temperature
  In this condition, we keep the DUTs warm for certain periods; for example, the temperature of the test is the same for one week.
2. **Second Condition:** Temperature Cycling
  Unlike the previous condition where the DUTs were continuously kept warm for a certain period, the DUTs in this condition will be heated cyclically for a certain period. In other words, in one week, we cool the DUTs daily and then bring them back to a high temperature. So far, no work has been found to compare the effects of cyclic temperature heating and constant temperature heating. Therefore, in this study, we will compare the effects of these two types of heating.
3. **Third Condition:** Room Temperature
  To evaluate the effects of heating, it is imperative to compare our results with a reference chip that is turned on permanently (during the test time) and at room temperature; thus, we added a third condition.

#### 3.2.2. Power Conditions

To conduct our experiment, we will focus on three main scenarios for power, which are described below.

1. **First Condition:** Power Off
  Here, the DUTs are not connected to any power source. Evaluating this condition is the main goal of our study.
2. **Second Condition:** Power On
  All of the DUTs in this condition are powered on. We use this condition as a reference, which means that the power-on condition can help us compare the results of this condition with the power-off condition.
3. **Third Condition:** Clock Freezing
  In this condition, we connect our DUTs to power, but the clock signal of the delay-based detector is frozen (i.e., the clock does not have an edge). Also, for the RO, the enable signal is equal to zero (i.e., the RO is inactive). We use this condition because the aging effect is sensitive to electrical stress. When there is no clock or it is inactive, then the detector is unable to detect an attack.

**Figure 6:**
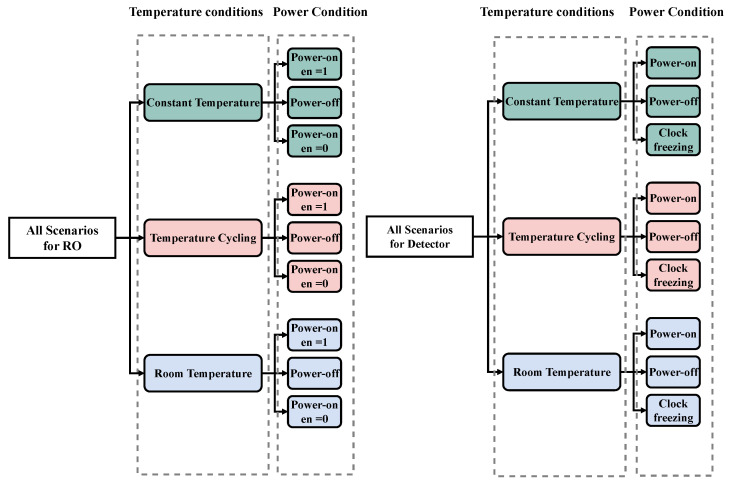
All of the scenarios for our experiment for DUTs (RO and detector).

Therefore, we have three temperature conditions for each detector and RO. Then, for each condition, we have three power conditions, which are shown in Figure 6.

### 3.3. Attacker Model

In this work, we consider an attacker who manipulates the circuit while it is powered off to alter the intrinsic characteristics of security sensors or attack detectors. The attacker could then successfully carry out an FIA when the circuit is powered on, exploiting the attack without detection by the compromised fault attack detector. Integrated circuits are particularly sensitive to heat, which alters the intrinsic electrical properties of transistors. Heating accelerates the aging of the circuit. It can be achieved with an oven, but local heating can also result from a laser attack.

For the scenarios outlined in Section 3.2, the attacker must physically access the target system to induce heating and disconnect its power supply to execute the powered-off attack. Additionally, for clock freezing, the attacker needs to freeze the clock of the detector to disable its oscillation.

## 4. Experiments

In this section, we present experiments conducted on actual devices to evaluate the impact of heating on the scenarios described in the previous section. We begin by describing the experimental setup, followed by the presentation of the experimental results for heating effects. Then, in the next section, these results will be discussed.

### 4.1. Experimental Setup

As mentioned in the previous sections, our objective is to evaluate the impact of overheating on the performance of the delay-based detector and the frequency of the RO in various power and temperature conditions. For each DUT (RO and delay-based detector), we examined nine scenarios: three temperature conditions and three power conditions. We implemented each scenario on a separate BASYS-3 board (manufactured by Digilent, Pullman, WA, USA) with an Xilinx Artix-7 FPGA (manufactured by AMD, Santa Clara, CA, USA), for a total of 18 boards.

#### 4.1.1. Measurement Setup

To evaluate the detector response time, we utilized an oscilloscope to measure the alarm signal’s activation time. For the RO, we measured the period with a 100 ps time resolution. It is important to mention that to determine the detection threshold accurately, we performed 10 successive measurements on each detector before averaging the results.

All measurements were taken with the chips at the same temperature, specifically normal room temperature. This requires removing the FPGAs from the climate test chamber and waiting for them to return to room temperature. Removing the DUT every day reduces the duration of exposure (about 8 h per day) and produces temperature cycles, with temperature rises and falls. However, state-of-the-art methods do not mention any particular effects in terms of aging caused by these cycles. Indeed, only the duration during which the components are exposed to high temperatures is known to produce aging effects. In addition to the experiments mentioned above, we conducted two additional tests to increase measurement accuracy: first, we evaluated the internal temperature of the chip and its effects on the RO frequency. This test helps determine if measurements need to be taken at a specific time after the chip is turned on. Secondly, we assessed how increasing internal temperature affects the RO frequency. Understanding this impact helps us gauge the importance of temperature on the detector threshold and identify potential measurement errors. To perform these tests, we accessed the internal temperature of the chips using the built-in temperature detectors in the Artix-7 FPGA.

#### 4.1.2. Delay-Based Detector Setup

First, we implemented the delay-based detector, as shown in Figure 4, using HDL. To evaluate delay-based detectors against power-off attacks, it is first crucial to ensure that the detector works correctly when the device is powered on. To achieve this, we performed an overclocking attack on the sensor while it was powered on. To perform an overclocking attack, we first changed the clock source from internal to external so that we could manipulate the clock frequency using a pulse generator. We used a Rigol DG4102 Waveform Generator to increase the circuit’s frequency. In normal mode, the detector’s operating frequency was 10 MHz, and when we increased the frequency from 10 MHz to 17.2 MHz, the alarm was triggered. This allowed us to validate the correct operation of the detector and determine its initial threshold detection frequency. To measure the thresholds of the alarm activation, we performed overclocking attacks on each detector and then measured the alarm activation signal with an oscilloscope.

As explained in the previous chapter, we have to evaluate three temperature conditions and three power conditions. Thus, for detector evaluation, we implemented each scenario with one BASYS-3, resulting in the use of nine boards in this study to assess detectors against POTA. To ensure a comprehensive evaluation, it was important to obtain a large number of results for each scenario, enhancing the reliability and accuracy of our tests. Therefore, rather than implementing just 1 detector per chip, we placed 27 detectors on each chip. This significantly increased the accuracy of our results. We implemented 27 detectors (ROs) in each BASYS-3 due to the limitation in the number of Pmod pins on the BASYS-3 board and because we aimed to realize this test manually. Since we have 27 detectors in each chip, each detector (RO) uses a different clock frequency. This approach is based on studies within the literature that showed different effects of heating on different RO frequencies [28]. Therefore, we can also evaluate the effects of heat and POTAs on different alarm frequencies. For this purpose, we implemented the detectors of each chip in three groups:

- **First Group:**
  Clock source: 25 MHz (T = 40 ns) Alarm activation with overclocking = 38–39 MHz
- **Second Group:**
  Clock source: 10 MHz (T = 100 ns) Alarm activation with overclocking = 19–21 MHz
- **Third Group:**
  Clock source: 2 MHz (T = 500 ns) Alarm activation with overclocking = 3–4 MHz

Since we have three different groups of detectors with different clock frequencies in each chip, we need three clock sources with different frequencies. For this, we used the Mixed-Mode Clock Management (MMCM) module, which is available on Artix-7 FPGA.

### 4.2. Ring Oscillator (RO) Setup

Our second DUT is the RO, as mentioned earlier. Therefore, like the detector, we also implemented it using HDL. To increase the number of results and ensure a comprehensive test, we implemented each scenario on an Artix-7 FPGA with 30 RO per FPGA. We divided 30 ROs into three groups with different frequencies:

- **First Group:** Frequency = 4 MHz
- **Second Group:** Frequency = 16 MHz
- **Third Group:** Frequency = 60 MHz

**Figure 7:**
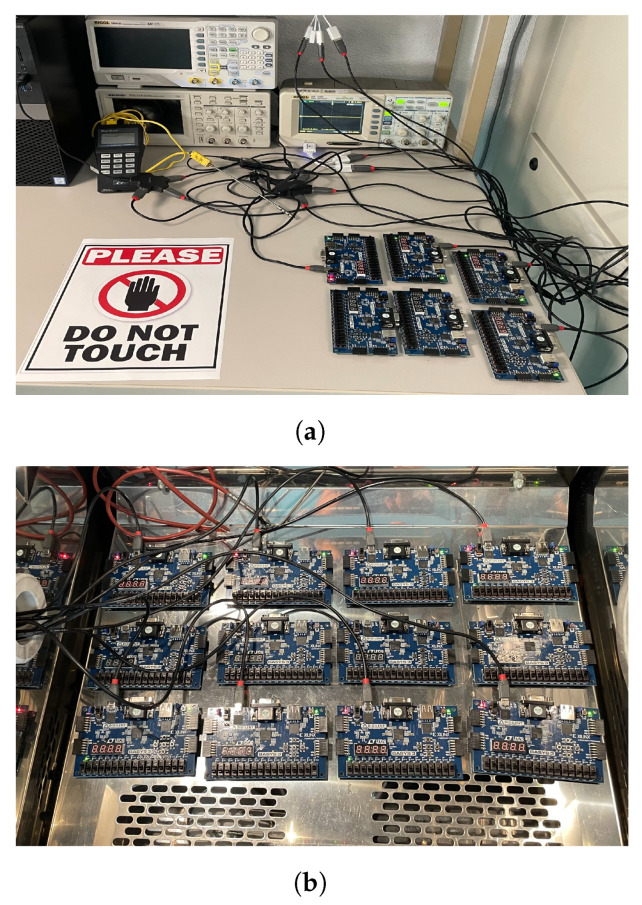
All our scenarios include temperature conditions (room temperature, temperature cycling, constant temperature) and power conditions (power on, power off, clock freezing). (**a**) Outside climatic room. (**b**) Inside climatic room.

To perform the practical evaluation, we used the Votsch VC 0018 climate test chamber, which can produce heat up to +95 °C, as shown in the figure. We subjected the delay-based detector and RO to various time cycles within the thermal chamber to examine the impact of heat on their performance. Figure 7 shows the general setup of our experiment; the chips that are inside the climate test chamber correspond to the constant temperature and temperature cycling scenarios, and the chips outside the room correspond to the room temperature scenario.

### 4.3. Experimental Results

In this section, the results of the tests described in the previous section will be presented. Table 1 shows how the RO frequency changes after it is turned on. These findings help us determine the optimal time to measure the RO frequency after activation. This matters because the frequency can vary after activation, affecting the accuracy of our main results.

Table 2 illustrates how varying the chip’s internal temperature affects different RO frequencies, with each operating at a unique frequency. As mentioned in the previous section, we measured the internal temperature using the temperature sensor available in the Artix-7 FPGA.

Table 3 and Table 4 display the results of the tests carried out over 21 days, with all measurements taken while chips were kept at room temperature. In these two tables, the variations in activation thresholds of the detectors and RO frequency are represented. As outlined in Table 3, the degradation in alarm activation showed notable variation across different temperature and power conditions. Under the power-on condition, the most significant degradation was recorded during the temperature cycling scenario, with a reduction of −1.98%. In contrast, under power-off conditions, the degradation rates for temperature cycling and constant temperature were comparable, showing slight increases of +0.36% and +0.31%, respectively. The most substantial overall degradation was observed under the clock-freezing with temperature cycling condition, where the degradation reached −2.53%, the highest among all scenarios evaluated. Similarly, the results presented in Table 4 highlight the degradation in RO frequency across the same set of conditions. In the power-on state with en = 1, the temperature cycling condition once again resulted in a considerable degradation, with a reduction of −1.75%. Under power-off conditions, both temperature cycling and constant temperature showed nearly identical degradation rates, with slight increases of +0.26% and +0.29%, respectively. Consistent with the trends observed for alarm activation. However, the most pronounced degradation in RO frequency was recorded in the power-on state with en = 0, where a substantial reduction of −2.03% was observed.

It is necessary to mention that all of the results obtained from the average of 27 detector activation threshold frequencies are the same for ROs. For an in-depth analysis, it is better to evaluate each group of frequencies separately.

Overall, +95 °C is the maximum temperature that the climate test chamber can deliver. It is lower than the temperature that a laser could achieve locally. However, in the context of this study, we want to see if we could observe a temperature attack effect when the circuit’s power is off. If we observe an effect by immersing the entire circuit in the climatic test chamber, a laser could certainly create the same variations, perhaps more quickly by applying higher temperatures.

In this section, we have detailed the complete setup process to be applied for our DUT, its implementation on the FPGA, and the specific details of the experimental arrangement. Additionally, we presented the results of two tests conducted to validate our measurements. In the first test, we measured the changes in the RO frequency from the moment it was activated up to 180 min later. This was carried out to determine whether the frequency remained stable over time. Our results showed that the frequency exhibited only minimal variations, which were negligible and had no significant impact on our overall findings. In our second test, we temporarily increased the internal temperature of the chip to ensure that a rise in temperature could indeed affect the RO frequency. This was completed to validate whether a temperature change directly impacts the frequency of ROs. Along with the results of our experiment on ROs and detectors, including the POTA outcomes, we will provide a comprehensive discussion and analysis of all the findings in the next section.

## 5. Discussion

The main goal of this chapter is to discuss all the results presented in the previous chapter. To make this easier to understanding, we divided these results into three groups:

The first group presents the results of measurement verification. This group comprises two evaluations: the first evaluates the percentage of changes in RO frequency after RO activation, and the second examines the effect of increasing the internal temperature on various RO frequencies. The second group presents the results of the delay-based detectors, while the final group displays RO outcomes. In the following sections, detailed explanations and insights into each category are provided.

### 5.1. Measurement Verification

#### 5.1.1. Percentage of RO Frequency Changes over Time After Activation

The objective of this evaluation is to determine the optimal timing for measuring the frequency of the RO after activation. The goal is to assess frequency variations within a specific time window after activation. By doing so, we aim to understand how the RO frequency stabilizes or changes over this period. This evaluation involves measuring the RO’s frequency repeatedly within the defined time frame to capture any trends or patterns that emerge. Ultimately, this analysis helps us pinpoint the most suitable moment for accurate frequency measurement after the RO’s activation, contributing to a better understanding of its behavior and performance characteristics.

**Figure 8:**
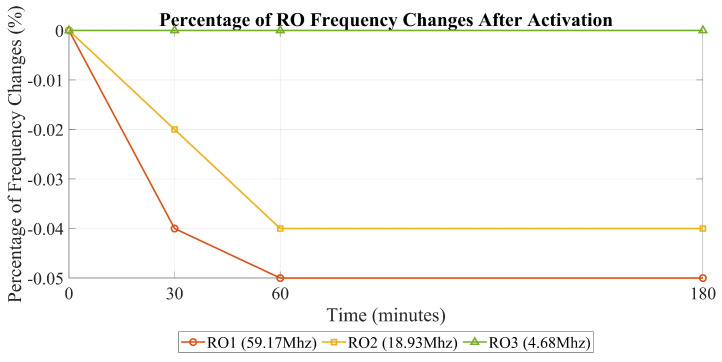
Percentage of RO frequency changes after activation.

In Figure 8, the degradation of the RO frequency is depicted from the moment of activation up to 180 min later. From this illustration, it becomes evident that the maximum degradation observed is smaller compared to the degradation observed in all heating scenarios. In simpler terms, this evaluation confirms the precision of our measurement.

Among all the heating scenarios (temperature cycling and constant temperature), the power-off scenario has the smallest effect on the RO, with a value of 0.26% in temperature cycling. The most substantial degradation after RO activation was 0.05%. This result indicates that the degradation resulting from the heating is greater than the measurement’s accuracy, reinforcing the reliability of this heating evaluation.

The results of this experiment help us determine whether the RO frequency remains stable after activation. This is crucial in understanding the optimal timing for our measurements. Our findings showed that the frequency remains consistent and does not change significantly, regardless of when we perform the measurements after the RO is activated. Therefore, the timing of our measurements does not impact the results.

#### 5.1.2. Effects of Internal Temperature on ROs with Different Frequencies

This evaluation was conducted to validate the impact of heating on our measurement approach by examining frequency degradation in relation to temperature increases. Our findings show a clear correlation between rising internal temperatures and frequency degradation. Specifically, the variations of 2–3% after heating (aging) are comparable to the effects of a +70 °C temperature increase. By allowing several hours to pass before taking measurements, we ensured that the observed degradation was not solely due to temporary internal temperature changes. Notably, the highest degradation observed was 7.98%, which exceeds all degradation values recorded for both the detector and RO under normal conditions. This discrepancy arises because our measurements were taken while the chip was still in its heated state, whereas previous evaluations of heating on the detector and RO were performed under normal conditions.

This distinction is critical for two reasons. First, if no degradation is observed while the chip is hot, we can reasonably assume that no degradation will occur once it cools down. However, in our case, significant variations were detected, confirming the presence of a heating effect. This evaluation improves our understanding of the relationship between temperature and frequency degradation in our measurement system.

**Figure 9:**
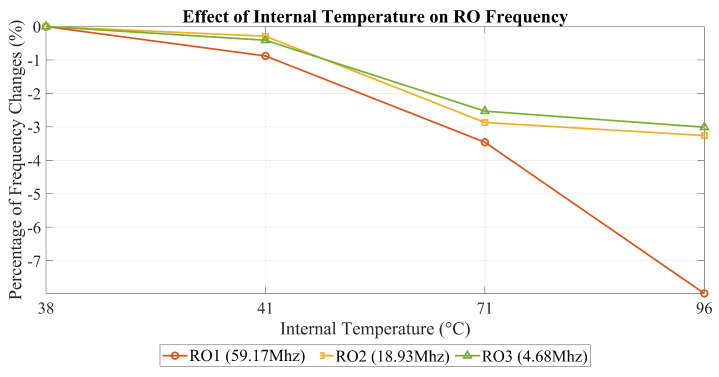
Impact of internal temperature changes on RO frequencies.

Additionally, Figure 9 illustrates the differential impact of heating on ROs operating at different frequencies. Three ROs are represented: the red RO at 59.17 MHz, the yellow RO at 18.93 MHz, and the green RO at 4.68 MHz.

In our experiment, we intentionally raised the internal temperature to +96 °C. At this elevated temperature, the red RO, operating at the highest frequency, experienced more degradation than the other two ROs. This visually confirms our earlier observation that heating affects ROs more significantly at higher frequencies than at lower ones.

### 5.2. Delay-Based Detector Analysis Against Heating

**Figure 10:**
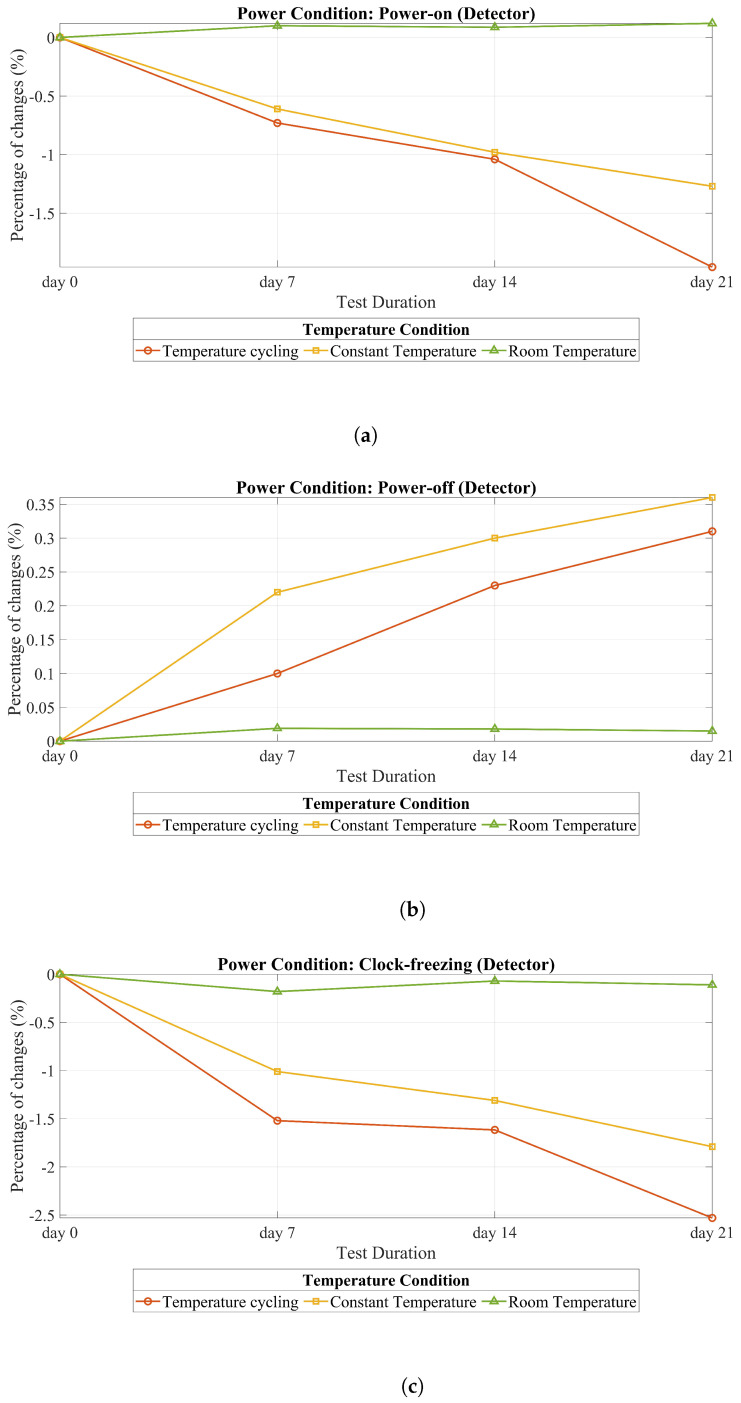
All of the temperature and power conditions for the delay-based detector. (**a**) Power on. (**b**) Power off. (**c**) Clock freezing.

As we explained earlier, we considered three different temperature conditions and three power conditions for the detector. Figure 10 shows all these scenarios for our detector. In Figure 10a, we can see the scenario where the detector is powered on under the three temperature conditions. Similarly, Figure 10b,c demonstrate the scenarios in which the detector is powered off and the clock is freezing, respectively.

In all power scenarios, room temperature serves as a useful reference point to compare the results of heating and non-heating conditions. As depicted in Figure 10b, when the circuit’s power is off, both the constant temperature and temperature cycling have a small impact. However, a comparison with the reference (room temperature) reveals an observable effect in the power-off scenario with constant and cycling temperatures. In the power-off attack scenario, the constant temperature has a greater impact than temperature cycling due to its sustained influence on the system. A constant temperature maintains a stable condition that can cause more pronounced and consistent changes in the circuit’s behavior during a power-off attack. Moreover, since no study has been carried out on POTAs, this issue may be related to the physical characteristics of the chip, which are out of the scope of this study. However, for other scenarios (i.e., power on and clock freezing), temperature cycling has a greater effect than a constant temperature.

As indicated in Table 3, implementing clock freezing across three temperature scenarios (room temperature, constant temperature, and temperature cycling) has a greater impact on the activation of the detector alarm compared to power-off and power-on conditions.

It appears logical and normal that the impact of heating during the power-off state is less than that of clock freezing. As explained earlier, we can simulate the heating effect through aging. The primary aging factor acting on the transistor is electrical stress. However, since there is no electrical stress on the transistor in the power-off state, this observation is expected.

In comparison to the power-on state, clock freezing has a more pronounced effect. This concept can be modeled using bias temperature instability (BTI) effects. As depicted in Figure 3, BTI involves two phases: stress and recovery. In the recovery phase, some of the degradation that occurred during the stress phase is recovered. In clock freezing, our clock remains constant, eliminating any clock edges and preventing oscillation between zero and one. Consequently, there is no switching for certain transistors. Therefore, we can conclude that aging in clock freezing results in more degradation compared to power-on conditions. Additionally, in the context of heating, it is observed that clock freezing has a more substantial effect than power-on conditions. This concept is shown in Figure 10a,c.

### 5.3. Ring Oscillator (RO) Analysis Against Heating

**Figure 11:**
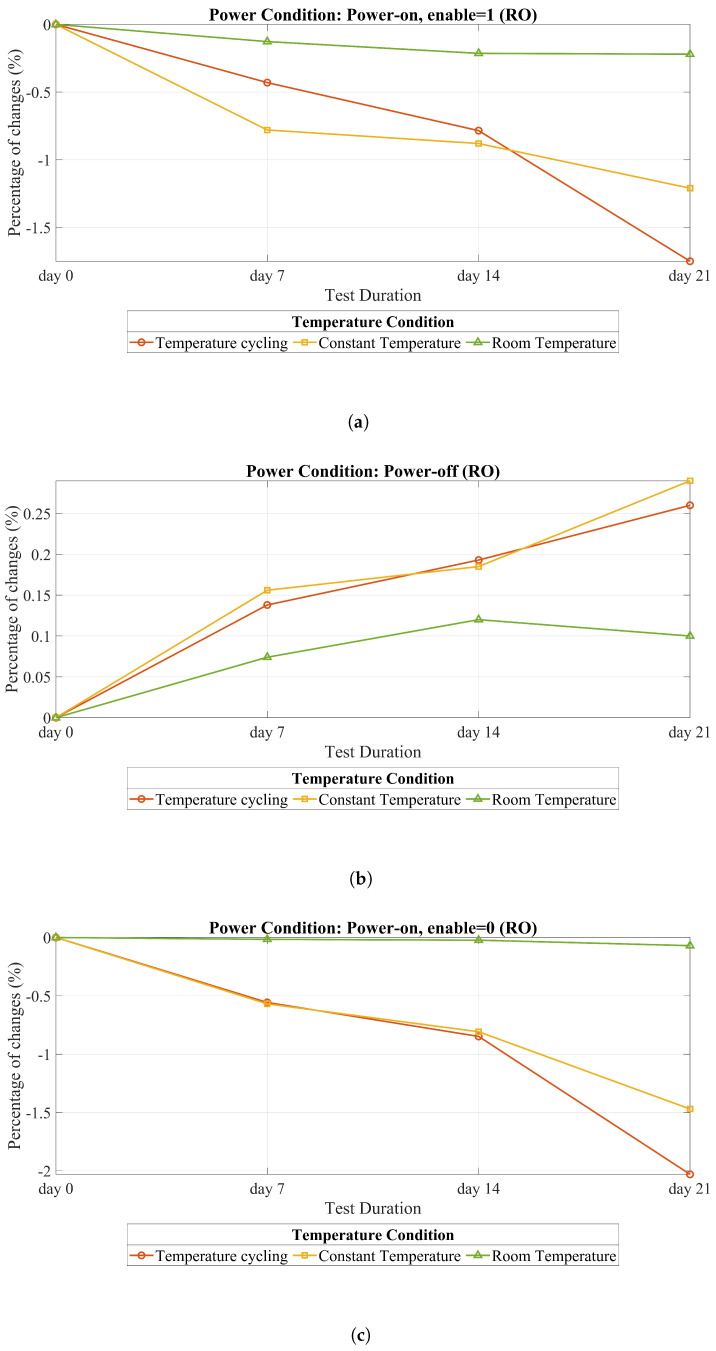
All of the temperature and power conditions for the RO. (**a**) Power on, enable = 1. (**b**) Power off. (**c**) Power on, enable = 0.

Figure 11 displays how heating over 21 days affects the RO. Notably, the figure reveals that the impact of POTAs on ROs is small but observable, similar to the detector. An important observation here is that clock freezing has a more significant impact on both the RO and detector compared to other scenarios. This is because when transistors remain in the same state for extended periods without switching between on and off states, they become more susceptible to aging, which can accelerate aging effects such as BTI. Consequently, we anticipate that clock freezing would have a greater effect on aging than scenarios involving power-on conditions (en = 1 in RO). In simpler terms, the freezing of the clock can intensify the aging process, leading to increased degradation compared to other power scenarios. Temperature cycling also leads to greater degradation in this context, mirroring the effects observed in the detector. This similarity in degradation could be attributed to the same underlying reasons observed in the case of the detector. As shown in [29], at a temperature of 90 °C (under DC stress and a 1.3 Vnom supply voltage), the RO frequency variation is around −3% after one year. Remarkably, after just one month, this variation is approximately −1.5%, which closely corresponds with our findings, despite the authors employing a higher power supply.

In this section, we talked about what we found in our experiments. We see that a power-on attack has a very small effect but an observable effect on the RO and detector. This type of attack can affect the characteristics of the chip, but we cannot find any previous works showing this effect. We also noticed something important about freezing the clock signal. Among all the situations we tested, freezing had the biggest effect. This was mainly because of some aging effects, such as BTI. We also found that when we heat the RO, the ones with higher frequencies are affected more compared to those with lower frequencies. Moreover, it is interesting that whether we measure the RO’s frequency right after we start it or after 180 min, the results are similar. This means the effects are consistent over time.

According to our result and attack model described in Section 3.3, Table 5 presents a comprehensive overview of our attack model from the perspective of the attacker. It outlines three key attack scenarios, power-on, power-off, and clock-freezing, detailing the capabilities required by the attacker for each scenario, along with the corresponding setup procedures. Additionally, the table examines the effects of these attacks on the detector and RO, providing insights into potential security threats posed by each attack type. Through meticulous analysis, the table underscores the critical importance of understanding and mitigating vulnerabilities in integrated circuits to safeguard against malicious exploits.

## 6. Conclusions

The objective of our study was to evaluate the effects of heating and POTAs on digital circuits and the susceptibility of delay-based and RO detectors. These types of attacks can jeopardize chip security since POTAs cannot be detected by active mechanisms.

In the case of a detector safeguarding a secure component, an attacker can manipulate either the circuits or the detector’s features by injecting faults (on permanent electrical parameters: for instance, delay modifications) when the chip is turned off. If the detectors are altered, then the attacker can perform other attacks while being undetected. Our investigation has demonstrated that POTAs can impact circuitry and detectors, leading to an increase in false negatives.

In a detector, if we can activate the alarm earlier than normal, this means that we increase false positives; when we activate the alarm after normal activation, this means that we increase false negatives. Increasing false-negative expansion, unlike increasing false positives, can cause security threats and attackers to enter the system; therefore, it is very important and fundamental to consider this type of attack. Our result shows that with clock freezing and power on, unlike POTAs, with heating, we can increase the false positive of delay-based detectors. This effect on delayed-based detectors will not create any security threat and can only increase false alarms with clock freezing and power on. In our future research, we intend to assess this type of attack for an extended period and on a wider variety of detectors. In particular, we will look for detectors impacted by POTAs and clock-freezing attacks to create false negatives.

Another potential future work for this study is the implementation of self-testing mechanisms. We have seen that a POTA has an effect, so we should protect our system against this type of attack by incorporating dedicated circuitry to check the integrity of the sensor after power-up. One way to achieve this is to use self-testing in our chip to check the characteristic variation every time after activation.

More specifically, within the context of the POP project, our plan is to use a laser and produce a dedicated test chip to conduct comparable experiments. These experiments will involve localized temperature attacks on various digital and analog circuits, including detectors. 

## Figures and Tables

**Figure 2:**
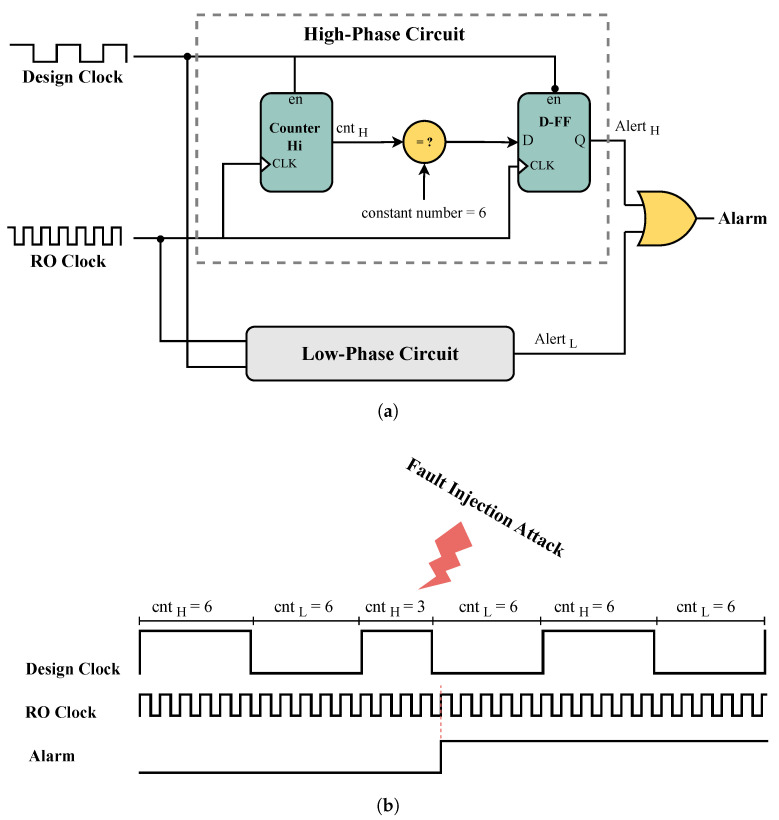
Schematic diagram and waveforms of the counter-based detector proposed in [20]. (**a**) Schematic of the counter-based detector. (**b**) The figure illustrates how FIA (marked by the red line) reduces the design clock period, decreasing the number of RO clock rising edges from 6 to 3 and activating the alarm.

**Table 1:** Percentage of RO frequency changes over time after activation.

Percentage of RO Frequency Changes After Activation (%)			
**Description of RO**	**After 30 min**	**After 60 min**	**After 180 min**
**RO1** (59.17 MHz)	−0.04 %	−0.05%	−0.05%
**RO2** (18.93 MHz)	−0.02 %	−0.04%	−0.04%
**RO3** (4.68 MHz)	0%	0%	0%

**Table 2:** Effects of internal temperature on ROs with different frequencies.

Impact of Internal Temperature Changes on RO Frequencies (%)				
**Description of RO**	**38 °C**	**41 °C**	**71 °C**	**96 °C**
**RO1** (59.17 MHz)	0 %	−0.88%	−3.46%	−7.98%
**RO2** (18.93 MHz)	0 %	−0.29%	−2.87%	−3.26%
**RO3** (4.68 MHz)	0 %	−0.41%	−2.53%	−3.01%

**Table 3:** Results of a heating delay-based detector.

Percent Change of Alarm Activation (%)			
**Temperature Condition**	**After 7 Days**	**After 14 Days**	**After 21 Days**
**Power Condition: Power−on**			
**Temperature Cycling**	−0.73%	−1.04%	−**1.98**%
**Constant Temperature**	−0.61%	−0.98%	−**1.27**%
**Room Temperature**	+0.10%	+0.08%	**+0.12**%
**Power Condition: Power−off**			
**Temperature Cycling**	+0.10%	+0.23%	**+0.31**%
**Constant Temperature**	+0.22%	+0.30%	**+0.36**%
**Room Temperature**	+0.019%	+0.018%	**+0.015**%
**Power Condition: Clock−freezing**			
**Temperature Cycling**	−1.52%	−1.61%	−**2.53**%
**Constant Temperature**	−1.01%	−1.31%	−**1.79**%
**Room Temperature**	−0.18%	−0.07%	−**0.11**%

**Table 4:** Results of a heating RO.

Percent Change of Ring Oscillator (RO) Frequency (%)			
**Temperature Condition**	**After 7 Days**	**After 14 Days**	**After 21 Days**
**Power Condition: Power−on, enable = 1**			
**Temperature Cycling**	−0.43%	−0.78%	−**1.75**%
**Constant Temperature**	−0.78%	−0.88%	−**1.21**%
**Room Temperature**	−0.127%	−0.214%	−**0.22**%
**Power Condition: Power−off**			
**Temperature Cycling**	+0.138%	+0.193%	**+0.26**%
**Constant Temperature**	+0.156%	+0.185%	**+0.29**%
**Room Temperature**	+0.07%	+0.12%	**+0.10**%
**Power Condition: Power−on, enable = 0**			
**Temperature Cycling**	−0.55%	−0.84%	−**2.03**%
**Constant Temperature**	−0.56%	−0.80%	−**1.47**%
**Room Temperature**	−0.01%	−0.02%	−**0.07**%

**Table 5:** Summary of our attack model from the attacker’s perspective.

Attacker Perspective			
	**Power-on**	**Power-off**	**Clock-freezing**
**Capabilities**	Requires physical access to the target and ability to heat it at constant or cycling temperatures.	Requires physical access to the target, a disconnected power supply, and ability to apply constant or cycling temperature.	Requires physical access to the target, the ability to freeze the clock, and the ability to apply constant or cycling temperature.
**Attack Setup**	Heating entire chip space, either maintaining a constant temperature of 96 °C or cycling between 0 °C and 96 °C over 21 days.	Heating entire chip space, either maintaining a constant temperature of 96 °C or cycling between 0 °C and 96 °C over 21 days.Turn off the target system.	Heating entire chip space, either maintaining a constant temperature of 96 °C or cycling between 0 °C and 96 °C over 21 days.Freeze the clock signal for the detector and disable oscillation for the RO.
**Effect on detector**	For temperature cycling and constant temperature, the alarm activation changes by −**1.98%** and −**1.27%**, respectively.	For temperature cycling and constant temperature, the alarm activation changes by **+0.31%** and **+0.36%**, respectively.	For temperature cycling and constant temperature, the alarm activation changes by −**2.53%** and −**1.79%**, respectively.
**Effect on RO**	Cycling and maintaining constant temperature can reduce the RO frequency by −**1.75%** and −**1.21%**, respectively.	Cycling and maintaining constant temperature can lead to an increase in the RO frequency by **+0.26%** and **+0.29%**, respectively.	Cycling and maintaining constant temperature can reduce the RO frequency by −**2.03%** and −**1.47%**, respectively.
**Security Threats**	This type of attack has the potential to increase false positives in delay-based detectors, resulting in early activation in comparison to normal functioning and increasing false alarms.The RO frequency decreases; for detectors that are based on the RO, it may cause security concerns.	This type of attack can **increase false negatives**, which means that the alarm can be active later than in normal mode, which is useful from the attacker’s point of view.The RO frequency decreases; for detectors that are based on the RO, this may cause security concerns.	This type of attack has the potential to increase false positives in delay-based detectors, resulting in early activation in comparison to normal functioning and increasing false alarms.The RO frequency decreases; for detectors that are based on the RO, it may cause security concerns.

## Data Availability

Data are contained within the article.
